# Research on Vibration Suppression Method Based on Double Loop Position Feedback Control

**DOI:** 10.3390/s26041244

**Published:** 2026-02-14

**Authors:** Yunfei Qu, Changhua Xu, Xin Zhang, Zhen Li, Hong Wang

**Affiliations:** 1 Shenzhen Inovance Technology Co., Ltd., Shenzhen 518000, China; quyunfei@inovance.com (Y.Q.); xuchanghua@inovance.com (C.X.); zhangxin@inovance.com (X.Z.); 2 The School of Mechanical Engineering and Automation, Harbin Institute of Technology, Shenzhen 518000, China; 24s153185@stu.hit.edu.cn

**Keywords:** double-loop position feedback control, linear extended state observer, velocity feedforward, vibration suppression, collaborative robots

## Abstract

**Highlights:**

**What are the main findings?**

- This study proposes a vibration suppression method combining double-loop position feedback control with a linear extended state observer (LESO) and speed feedforward.
- Simulation results demonstrate that the method effectively suppresses mechanical resonance (reducing speed fluctuations by 84.4%) and eliminates steady-state position tracking errors.
- Experimental verification on a collaborative joint platform further confirms that the proposed strategy significantly attenuates load speed vibration and validates its feasibility in practical engineering applications.

**What are the implications of the main findings?**

- The proposed strategy successfully balances the stability of semi-closed-loop control with the high accuracy of full-closed-loop control for collaborative robot joints.
- It provides a practical engineering solution for mitigating nonlinear effects (elasticity and backlash) in harmonic-reducer-based servo systems.

**Abstract:**

Aiming at the problem that the position control accuracy of the traditional semi-closed-loop control and the vibration caused by the nonlinear characteristics of the system are easily affected by the full closed-loop control, a double-loop position feedback control based on the state information feedback of the motor and the load is proposed. Based on the double-loop position feedback control framework, a vibration suppression method combining the linear extended state observer, torque feedback compensation and speed feedforward is introduced. The simulation results show that the proposed control method effectively suppresses load vibration, improves the system’s servo control performance, and maintains position control accuracy.

## 1. Introduction

The joint servo drive system of collaborative robots typically consists of motors, brakes, harmonic reducers, and encoders. Collaborative robots usually come with additional built-in sensing devices, such as a dual encoder configuration, whereas industrial robots do not. This design enables simultaneous use of status information from both the motor and load sides, thereby improving servo control performance [1,2]. Semi-closed-loop position control that feeds back the corner information of the motor end is the most commonly used control mode. In semi-closed-loop control, because it lacks corner information feedback from the load end, the transmission from the motor to the load operates in open-loop. The nonlinear characteristics of the harmonic reducer itself cause angular transmission errors [3]. To improve position control accuracy, full-closed-loop control usually replaces semi-closed-loop control. Full-closed-loop control uses a linear encoder at the load end to send factual angle errors back to the position controller, thereby improving position control accuracy. However, full-closed-loop control also accounts for the system’s nonlinear features, such as elasticity, backlash, and angle transmission errors, in the closed-loop control. So this reduces servo performance. The double-loop position feedback control designed in this paper combines the stability of semi-closed-loop control and the position control accuracy of full-closed-loop control to achieve an ideal control effect.

Because the actual transmission devices in servo drive systems are not ideal rigid bodies, they have some elasticity. This inherent elasticity increases the likelihood of mechanical resonance during actual operation, causing not only noise pollution but also severe damage to mechanical transmission devices, thereby impairing their service life [4]. Therefore, vibration suppression is important for improving the control performance of servo systems and ensuring their safe and stable operation [5,6].

Relevant literature generally divides vibration suppression methods for servo drive systems into two categories: passive and active. Passive suppression methods include trajectory planning on the input position reference [7], input shaping algorithms [8,9], and filter-based compensation. Recently, new methods for trajectory planning have been proposed, further reducing system resonance by optimizing trajectory smoothness and limiting acceleration [10,11,12]. Among passive methods, researchers widely employ notch filter techniques [4] to mitigate motor current oscillations and suppress electromagnetic torque fluctuations. Adaptive notch filters [13] use the Fast Fourier Transform (FFT) to identify resonant frequencies and automatically adjust their parameters. However, researchers typically use notch filters in semi-closed-loop systems; in full-closed-loop systems with lower oscillation frequencies, they may degrade performance. Furthermore, they cannot handle gap nonlinearities, impose heavy computational loads, and may introduce phase lag that harms stability.

Regarding active suppression, relevant literature has explored various strategies. Reference [14] used the pole placement method to cancel zero-poles and mimic a rigid system, though the calculation is complex. The singular perturbation method [15] splits the system into fast and slow subsystems to suppress vibration, but it often lacks robustness and stability proof. $H_{\infty }$ control [16] utilizes sensitivity functions to improve tracking and robust stability, yet selecting the appropriate weighting function remains a primary challenge.

Furthermore, researchers actively investigate vibration suppression using a double-loop position feedback control framework. However, existing methods still face significant challenges. Reference [17] introduced an adaptive recoil compensation technique within a full-closed-loop framework, though stability guarantees are difficult to provide. State feedback methods [18,19] offer flexible pole allocation, but cascaded structures may reduce bandwidth. To address complex nonlinearities like friction and elasticity, extended state observers (ESOs) [20,21] have been employed. While effective, conventional nonlinear ESOs lack systematic parameter tuning rules. Although the linear extended state observer (LESO) [22,23] simplifies parameter tuning while maintaining robustness, a rigorous theoretical framework for integrating it with a double-loop control system to decouple stability and accuracy remains needed.

To address these challenges, this paper proposes a vibration suppression strategy. The approach uses double-loop position feedback, LESO, and velocity feedforward. The double-loop structure acts as a frequency-domain complementary filtering mechanism. Unlike conventional compensation techniques, this mechanism shows clearly how the time constant T balances low-frequency precision and high-frequency stability. It also ensures robust stability through frequency-domain decoupling. This strategy establishes a composite framework for disturbance rejection. In this framework, the LESO, configured via bandwidth parameterization, actively estimates and compensates for nonlinear lumped disturbances that the linear loop cannot handle. Compared to traditional cascaded feedback and nonlinear observers, this strategy maintains high system bandwidth by using velocity feedforward. It also significantly simplifies engineering implementation through the “3w” tuning method.

Following the introduction, Section 2 analyzes the mechanical resonance mechanism and the system’s dynamic model. Section 3 discusses the limitations of traditional loops, which motivates the composite strategy detailed in Section 4. Section 5 and Section 6 present the simulation and experimental results. Finally, Section 7 concludes the paper.

## 2. Resonance Analysis of Servo Drive Systems

**Figure 1:**
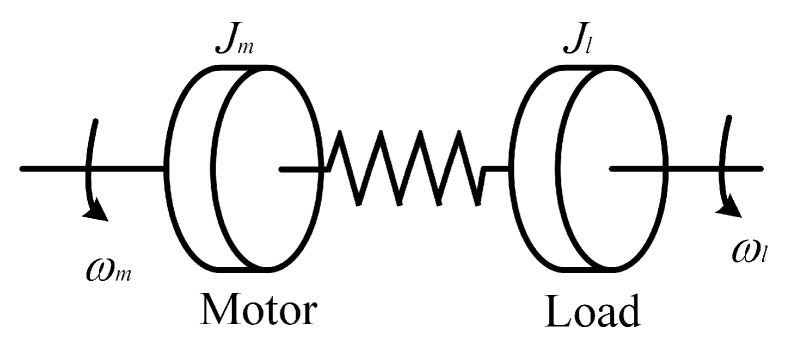
Schematic diagram of the two-inertia structure.

Figure 1 simplifies the joint servo drive module to a dual inertia system. A transmission shaft connects the motor and the load. The transmission shaft has a certain elasticity, with a stiffness of $K_{s}$. When the transmission shaft twists, it generates a torque $T_{s}$. For the motor, this torque is its load torque; for the load, it is the driving torque.

**Figure 2:**
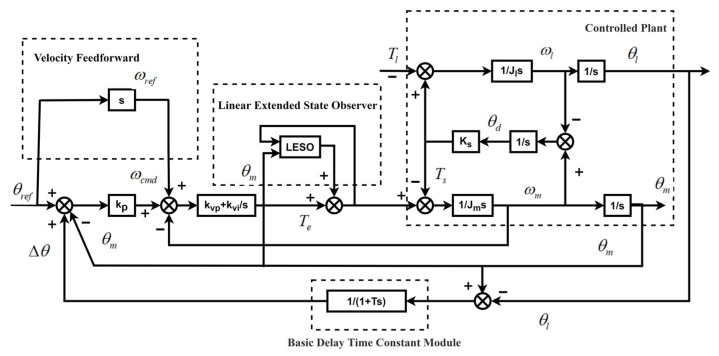
Control block diagram of the dual inertia system.

Analysis of Figure 1 leads to the control block diagram shown in Figure 2.

Here, $\theta_{ref}$ is the reference value of the motor angle. $\theta_{m}$ and $\theta_{l}$ denote the motor angle and the load angle, respectively. $\omega_{m}$ and $\omega_{l}$ are the motor speed and the load speed, respectively. $\omega_{ref}$ and $\omega_{cmd}$ represent the motor speed reference and the motor speed command, respectively. $T_{l}$, $T_{s}$ and $T_{e}$ are the load torque, transmission shaft torque and motor torque, respectively. $J_{l}$ and $J_{m}$ stand for the load inertia and motor inertia, respectively. $K_{s}$ is the shaft elastic coefficient. T is the time constant. $k_{p}$ is the proportional coefficient of the position loop. $k_{vp}$ and $k_{vi}$ are the proportional coefficient and integral coefficient of the speed loop, respectively.

Considered as a dual inertia system, the controlled object is governed by the following differential equations:


(1) $$\left\{\begin{array}{l} J_{m}{\dot{\omega }}_{m}=T_{e}-T_{s}\\ J_{l}{\dot{\omega }}_{l}=T_{s}-T_{l}\\ T_{s}=K_{s}(\theta_{m}-\theta_{l})\\ \omega_{m}={\dot{\theta }}_{m}\\ \omega_{l}={\dot{\theta }}_{l} \end{array}\right.$$

${\dot{\omega }}_{m}$ is the derivative of $\omega_{m}$, and ${\dot{\omega }}_{l}$ is the derivative of $\omega_{l}$. ${\dot{\theta }}_{m}$ is the derivative of $\theta_{m}$, and ${\dot{\theta }}_{l}$ is the derivative of $\theta_{l}$. The transfer function from motor torque to motor speed is as follows:


(2) $$G_{1}(s)=\frac{\omega_{m}}{T_{e}}=\frac{J_{l}s^{2}+K_{s}}{J_{m}J_{l}s^{3}+s(J_{m}+J_{l})K_{s}}$$

The transfer function representing the transmission from motor torque to load speed is as follows:


(3) $$G_{2}(s)=\frac{\omega_{l}}{T_{e}}=\frac{K_{s}}{J_{m}J_{l}s^{3}+s(J_{m}+J_{l})K_{s}}$$

By analyzing the characteristic equation of Equation (2), the system’s dynamical roots can be separated. There is a rigid-body mode, which corresponds to the pole at the origin, and a flexible oscillation mode, which corresponds to the conjugate imaginary poles. This flexible mode determines the resonant frequency $f_{NTF}$. Additionally, the anti-resonant frequency $f_{ARF}$ arises from the zeros of the transfer function, where the load’s inertial torque cancels the spring torque. Thus, the expressions are derived as follows:


(4) $$\left\{\begin{array}{c} f_{ARF}=\frac{1}{2\pi }\sqrt{\frac{K_{s}}{J_{l}}}Hz\\ f_{NTF}=\frac{1}{2\pi }\sqrt{K_{s}(\frac{1}{J_{m}}+\frac{1}{J_{l}})}Hz \end{array}\right.$$

When the system frequency reaches the anti-resonance frequency, the energy input to the motor immediately flows into the load. This causes energy to be transferred between the load’s kinetic energy and the shaft’s elastic potential energy, leading to load vibration. Especially in systems with a large load inertia and a small elastic coefficient, the anti-resonance frequency is low and falls within the system control bandwidth. This is one of the reasons why collaborative robots vibrate during uniform low-speed motion.

## 3. Characteristics of Semi-Closed and Full-Closed Loop Control

**Figure 3:**
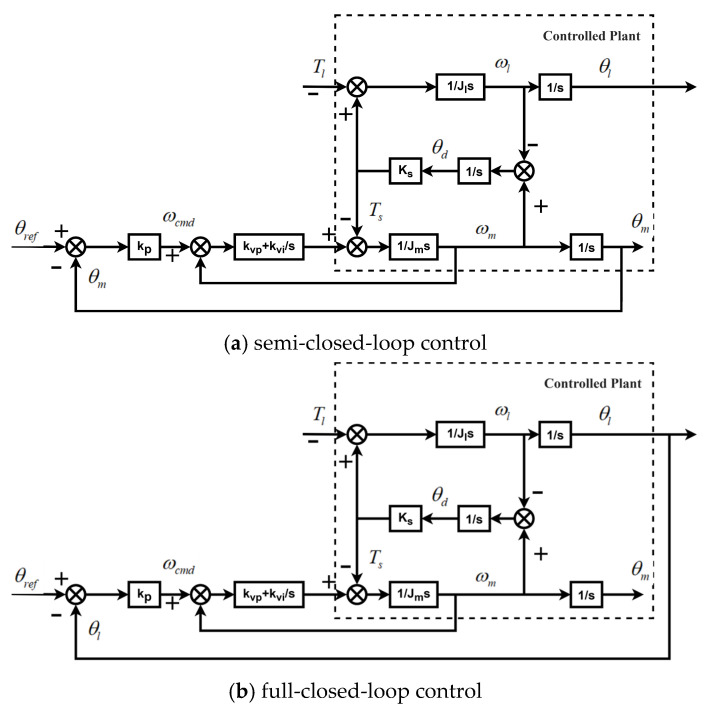
Position control framework.

Figure 3 shows the cascaded framework based on semi-closed-loop and full-closed-loop position control. The position loop uses P (proportional control), and the speed loop uses PI (proportional–integral control). Figure 3a realizes position control by feeding back the angular information of the motor shaft. Semi-closed-loop control achieves stable servo drive but results in a significant position error. To achieve more precise position control, angular information at the load end is fed back to the controller. This method is called full-closed-loop control.

**Figure 4:**
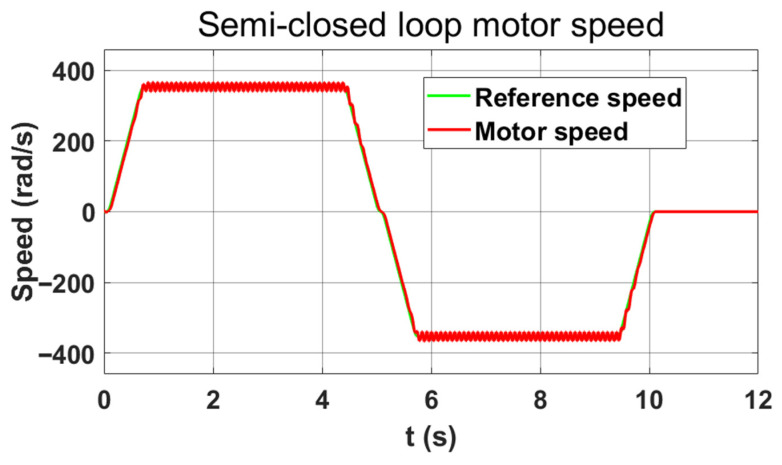
Semi-closed loop control motor speed curve.

**Figure 5:**
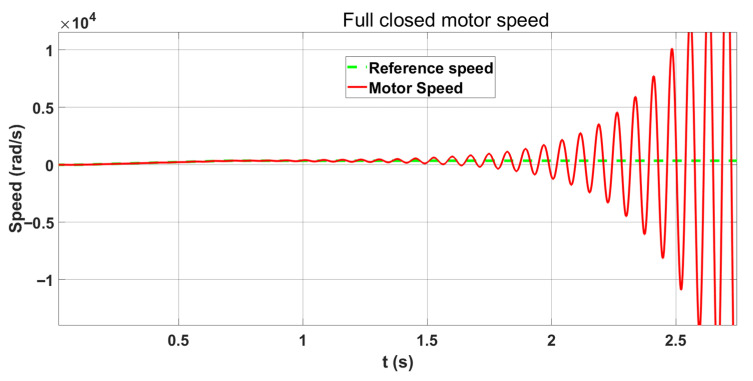
Motor speed curve for full closed-loop control.

Figure 4 illustrates the motor speed response to a reference command under semi-closed-loop control. The fluctuations in the curve are due to the inclusion of nonlinear characteristics, such as backlash and angle transmission errors, in the simulation model. Although the motor speed fluctuates in the figure, the entire process remains stable. Figure 5 shows that the system diverges when using the same position and speed loop gains under full-closed-loop control.

To compare the performance of semi-closed-loop control and full-closed-loop control, this paper draws the Bode plot of the transfer function from torque $T_{e}$ to load speed $\omega_{l}$.

**Figure 6:**
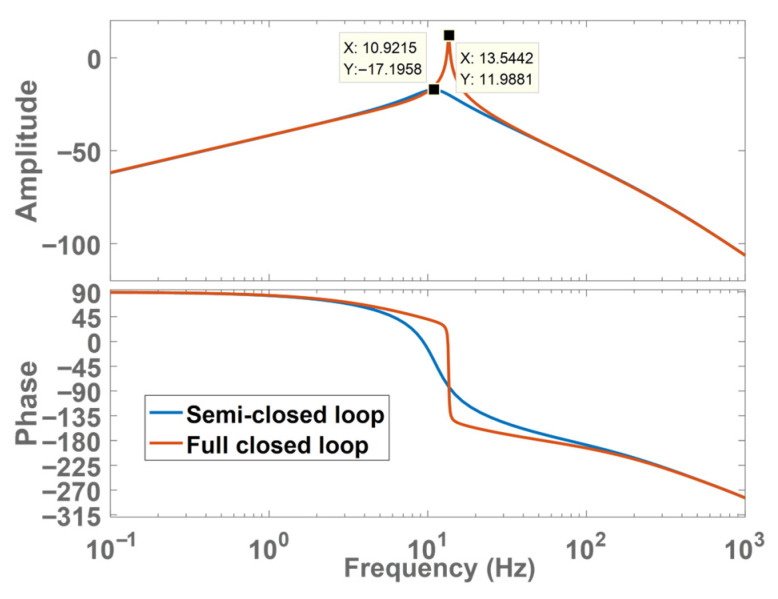
Bode plot of torque to load speed.

From the Bode plot in Figure 6, the amplitude–frequency margin of full-closed-loop control is lower than that of semi-closed-loop control. This is why the system diverges under full-closed-loop control when the position and speed loop gains are equal.

As shown in the control block diagram in Figure 2, semi-closed-loop and full-closed-loop control are combined by adding a basic delay-time constant module. Modifying the time constant adjusts the weights of the semi-closed-loop and full-closed-loop control in the double-loop position feedback. Let $\theta_{m}$ and $\theta_{l}$ denote the motor and load positions, respectively. The synthesized feedback position $\theta_{feed}$ is defined as:


(5) $$\theta_{\mathrm{feed}}(s)=-\theta_{m}(s)-\frac{1}{1+Ts}[\theta_{l}(s)-\theta_{m}(s)]$$

Rearranging the terms reveals the complementary filter structure:


(6) $$\theta_{\mathrm{feed}}(s)=-(1-\frac{1}{1+Ts})\theta_{m}(s)-\frac{1}{1+Ts}\theta_{l}(s)$$

The implementation principle is as follows:

- When the time constant T→∞, the low-pass filter gain approaches 0. $\theta_{feed}=\Delta \theta -\theta_{m}=0*(\theta_{m}-\theta_{l})-\theta_{m}=-\theta_{m}$, this situation is equivalent to semi-closed-loop control.
- When the time constant T→0, the low-pass filter gain approaches 1. $\theta_{feed}=\Delta \theta -\theta_{m}=1*(\theta_{m}-\theta_{l})-\theta_{m}=-\theta_{l}$, In this case, it is equivalent to full-closed-loop control. $\theta_{feed}$ is the final feedback angle.

Therefore, by selecting an appropriate time constant T, the double-loop position feedback control can combine the stability of semi-closed-loop control and the position control accuracy of full-closed-loop control to achieve the ideal control effect.

**Figure 7:**
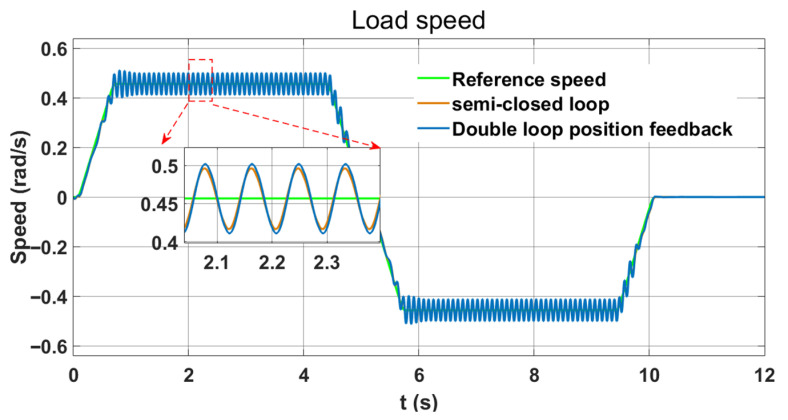
Semi-closed loop and double-loop position feedback load speed curves.

**Figure 8:**
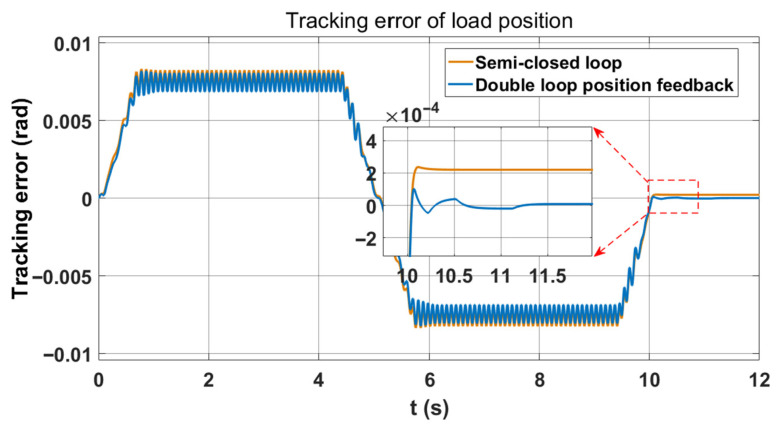
Semi-closed loop and double-loop position feedback load position tracking error curves.

Next, the following section compares the performance between double-loop and semi-closed-loop position feedback. Since robots may exhibit low-speed jitter during operation, this paper focuses on evaluating the speed and position tracking accuracy of the end load. Figure 7 demonstrates that double-loop position feedback control guarantees system stability, with only a slight increase in speed fluctuation at the load end compared to semi-closed-loop control. Furthermore, the position tracking error curve in Figure 8 indicates that double-loop position feedback control can effectively reduce the static position tracking error associated with semi-closed-loop control.

## 4. Vibration Suppression Methods—Analysis and Design

From the analysis in Section 3, the double-loop position feedback method can improve position control accuracy while maintaining system stability. However, the vibration speed at the load end is still relatively high. Based on double-loop position feedback control, this study proposes using a linear extended state observer to detect torque fluctuations caused by the system’s nonlinear characteristics. It then performs torque compensation to suppress mechanical resonance. At the same time, combined with speed feedforward, it improves system response and reduces steady-state position following error.

### 4.1. Torque Compensation Control Based on LESO

The extended state observer (ESO) is a core component in active disturbance rejection control. It can work without a mathematical model of the controlled object, treating model uncertainties and external disturbances as lumped disturbances. It then expands these disturbances into a new state variable for observation. Nonlinear extended state observers contain complex nonlinear functions. Their parameter tuning is difficult and lacks sufficient theoretical support. In Reference [24], a parameter configuration method for the linear extended state observer (LESO) was proposed by linearizing and parameterizing the nonlinear ESO. Only one parameter needs adjustment, making it more suitable for engineering applications.

In the design of LESO, the motor speed $\omega_{m}$ is selected as the output quantity y, and the electromagnetic torque $T_{e}$ is chosen as the input quantity u. Thus, the relationship between the electro- magnetic torque and the motor speed can be described as a second-order dynamic system:


(7) $$\left\{\begin{array}{l} {\dot{x}}_{1}(t)=x_{2}(t)\\ {\dot{x}}_{2}(t)=f(t,x_{1}(t),x_{2}(t),w(t))+bu(t)\\ y(t)=x_{1}(t) \end{array}\right.$$

In the equation, $b=\;1/J_{m}$ is the control gain. $f(t,x_{1}(t),x_{2}(t),w(t))$ is the sum of torque disturbance from nonlinear characteristics and external disturbance, expressed as the expanded state $x_{3}$. Thus, system (5) can be rewritten as:


(8) $$\left\{\begin{array}{l} {\dot{x}}_{1}(t)=x_{2}(t)\\ {\dot{x}}_{2}(t)=x_{3}(t)+bu(t)\\ {\dot{x}}_{3}(t)=h(t)\\ y(t)=x_{1}(t) \end{array}\right.$$

Here, h(t) represents the derivative of the lumped interference.

Constructing an observer in the form of Luenberger:


(9) $$\left\{\begin{array}{l} \dot{\hat{x}}=A\hat{x}+Bu+L(y-\hat{y})\\ \hat{y}=C\hat{x} \end{array}\right.$$

Here


$$\begin{array}{c} A=\left[\begin{array}{c} 0\;1\;0\\ 0\;0\;1\\ 0\;0\;0 \end{array}\right],B=\left[\begin{array}{c} 0\\ b\\ 0 \end{array}\right],C=\left[1\;0\;0\right],\\ L=\left[\beta_{1}\;\beta_{2}\;\beta_{3}\right] \end{array}$$

Since the LESO is a linear model, its parameters can be configured in the frequency domain using the bandwidth parameterization technique [24]. This paper adopts a pole placement strategy, referred to here as the “3w” method, to configure the parameters. By placing all the observer poles at the same bandwidth $-w_{0}$, the desired characteristic polynomial expands to ${\left(s+w_{0}\right)}^{3}=s^{3}+3w_{0}s^{2}+3w_{0}^{2}s+w_{0}^{3}$. Matching the coefficients with the observer characteristic equation, the gains are determined as follows:


(10) $$\beta_{1}=3w_{0},\beta_{2}=3w_{0}^{2},\beta_{3}=w_{0}^{3}$$

Here, $w_{0}$ is the bandwidth. Subtract the original system from the observer, which yields


(11) $$\dot{e}=A_{e}e+Eh$$

Here


$$A_{e}=A-LC=\left[\begin{array}{c} -\beta_{1}\;1\;0\\ -\beta_{2}\;0\;1\\ -\beta_{3}\;0\;0 \end{array}\right],\;E=\left[\begin{array}{c} 0\\ 0\\ 1 \end{array}\right]$$

The characteristic polynomial of the $A_{e}$ matrix is $f(\lambda )=\lambda^{3}+\beta_{1}\lambda^{2}+\beta_{2}\lambda +\beta_{3}$. To ensure observer error convergence, all eigenvalues should lie in the left-half plane. Using the specified configuration parameters gives $f(\lambda )=(\lambda +w_{0}{)}^{3}$. With positive bandwidth $w_{0}$, all eigenvalues will be in the left half-plane. Selecting an appropriate bandwidth $w_{0}$ allows the linear extended state observer to estimate the total disturbance.

### 4.2. Speed Feedforward Design

**Figure 9:**
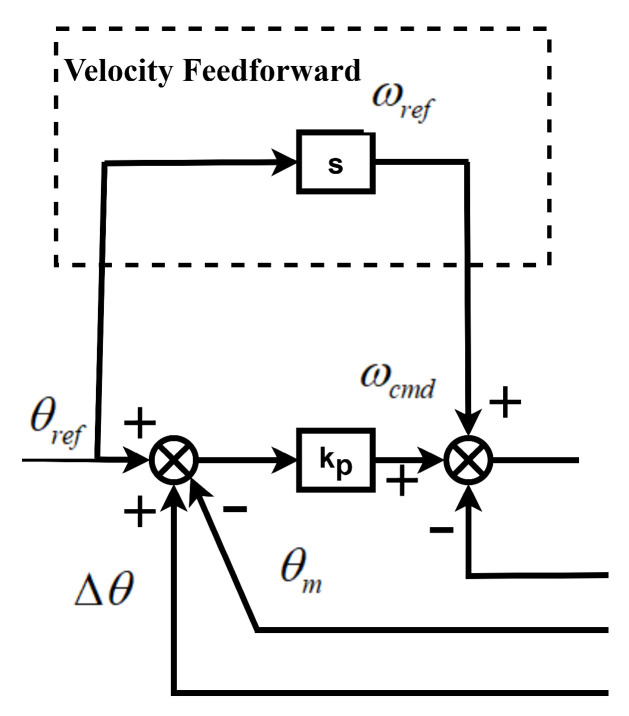
Velocity feedforward.

The speed feedforward processing method, as shown in Figure 9, differentiates the position command to obtain the speed feedforward command. Adding the feedforward link can improve command response and enhance the command loop bandwidth. However, it does not affect the stability margin or the disturbance response. With the feedforward method, the command response is not constrained by the control law. Since the feedforward gain does not form a loop, it does not compromise system stability. Adding feedforward increases the bandwidth of the command response but will slightly raise the peak.

## 5. Analysis of Simulation Results 

To verify the validity of the theoretical analysis, this study compared the control algorithm designed in this article with the traditional cascade PI control algorithm. This study establishes a double-loop position feedback servo control simulation system in MATLAB/Simulink, whose control block diagram shown as Figure 2. Nonlinear characteristics of the system, such as clearance and angle transmission errors, were incorporated into the simulation model. Table 1 presents the designed operating conditions, and the electromechanical parameters are as follows.

In addition, we also considered the notch filter method from Reference [4] for comparison, with its equation form as follows:

(12) $$F_{n}(s)=\frac{s^{2}+2\zeta_{2}\omega_{f}s+\omega_{f}^{2}}{s^{2}+2\zeta_{1}\omega_{f}s+\omega_{f}^{2}}$$

where $\zeta_{1}$ and $\zeta_{2}$ are coefficients of the filter, and are designed on depth and width of the notch filter. Natural frequency of the filter is denoted by $\omega_{f}$. After repeated attempts, we determine an optimal set of notch filter parameters, as shown in Table 1.

**Figure 10:**
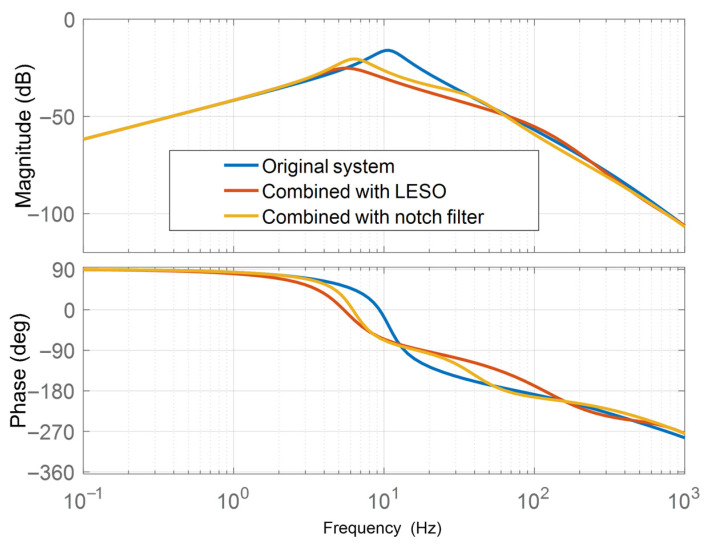
Bode plot of using the LESO system.

Figure 10 shows that employing LESO shifts the amplitude–frequency characteristic curve downward at the original system’s resonant frequency, effectively suppressing mechanical resonance.

**Figure 11:**
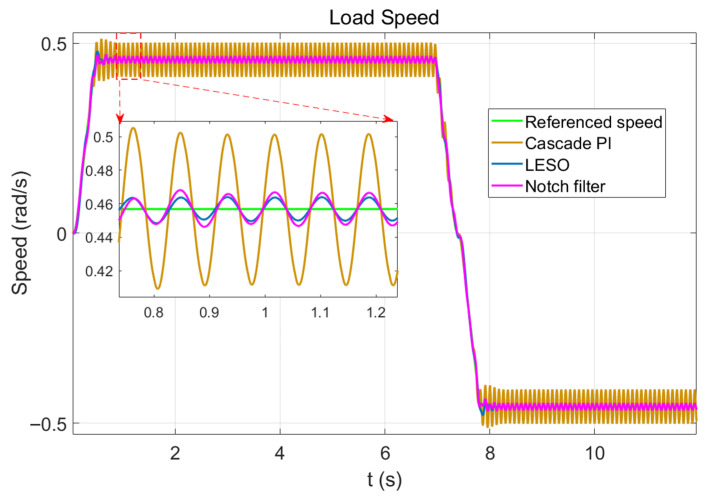
Effect of LESO vibration suppression.

Figure 11 shows that adding LESO significantly enhances vibration suppression compared to using only series PI. The speed fluctuation at the constant-speed end decreases by 84.4%, demonstrating the vibration suppression efficacy of LESO. In comparison, under the same conditions, the notch filter method can reduce speed fluctuation by 76.6%. Furthermore, as shown in the Bode diagram, the stability of the notch filter is also weaker than LESO.

**Figure 12:**
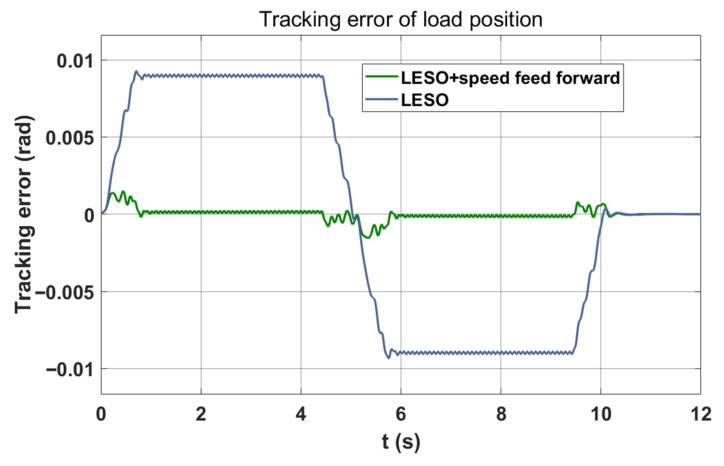
LESO combined with velocity feedforward load position tracking error curve.

In Figure 12, incorporating speed feedforward eliminates the position tracking error during the constant-speed segment. At this juncture, the proportional coefficient of the speed feedforward is set at 0.25.

## 6. Experimental Verification

**Figure 13:**
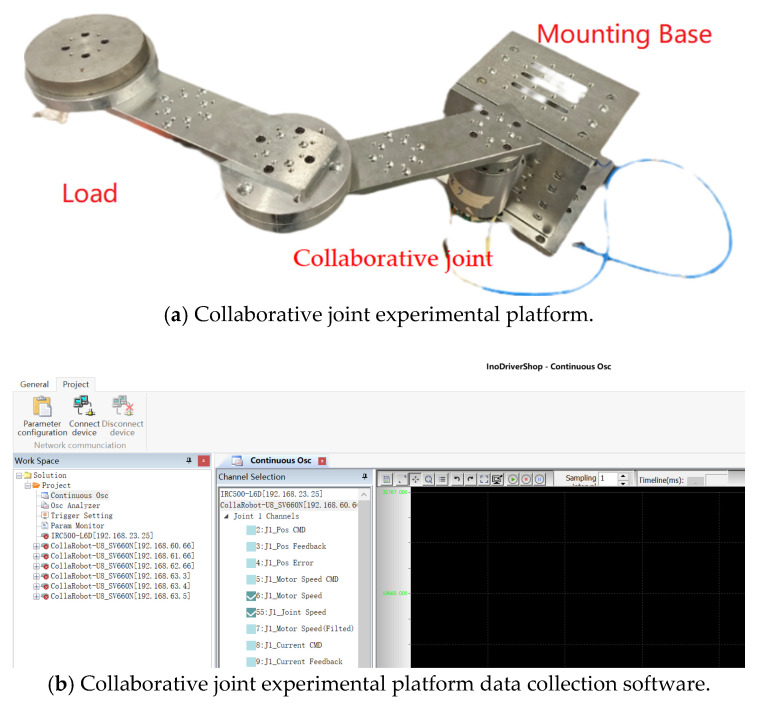
Collaborative joint experimental platform.

While Section 5 validated the effectiveness of the vibration suppression method through simulation, this section provides experimental verification by building a collaborative joint simulation platform. Figure 13 shows the collaborative joint experimental platform, which simulates a load by adding a two-link mechanism. The end flange can be loaded multiple times to verify extreme working conditions.

**Figure 14:**
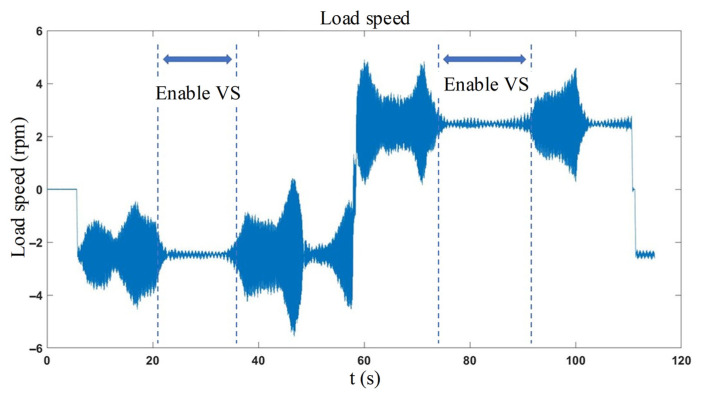
Vibration suppression enabled during part of uniform motion.

The experimental setup features four 2 kg loads at the ends (with each link weighing 3 kg) and a motor speed command of 200 rpm (corresponding speed at the load end is approximately 2.4691 rpm). Figure 14 demonstrates the vibration suppression activated during the uniform forward and reverse rotation. Notably, the dashed line segment highlights a significant reduction in the speed curve’s vibration.

**Figure 15:**
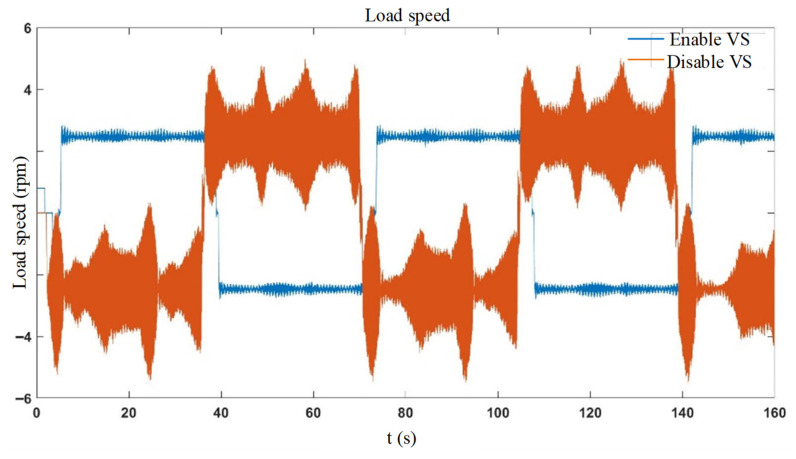
Full process vibration suppression enabled/disabled.

Figure 15 compares the end speed vibration with vibration suppression fully disabled (red curve) and fully enabled (blue curve). The results demonstrate that enabling vibration suppression significantly attenuates speed vibration.

## 7. Conclusions

This paper addresses the mechanical resonance suppression issue in harmonic-drive servo systems, which are gear mechanisms commonly used in precise motion control. We analyzed the dynamic coupling of the dual inertia system and proposed a composite control strategy. The key conclusions are summarized as follows:

- The double-loop structure functions as a frequency-domain complementary filter, resolving the trade-off between stability margin (mainly by the semi-closed loop) and positioning accuracy (mainly by the full-closed loop) by tuning the time constant T.
- The LESO, configured using the bandwidth parameterization method (which sets how quickly the observer responds to changes), provides a theoretical approach to address complex nonlinearities. These nonlinearities, such as backlash (lost motion due to mechanical link gaps) and friction (resistance to motion), are estimated as combined disturbances. This method increases robustness to changes in system parameters.
- Simulation results demonstrate that the proposed method reduces speed fluctuations by 84.4% and completely eliminates steady-state position errors. Experimental results confirm that vibration amplitude, measured as the peak deviation from the mean position, is markedly lower than with traditional PI control. Collectively, these results decisively demonstrate the method’s feasibility and effectiveness for engineering applications.

Future work will extend this vibration suppression strategy from a single joint to multi-degree-of-freedom (multi-DOF) collaborative robots by rigorously investigating joint-coupling effects. In parallel, we will implement adaptive parameter tuning algorithms to ensure robust control performance under varying load conditions.

## Figures and Tables

**Table 1:** Simulation parameters of servo control.

Variable Name	Parameter Value
Torque Constant/(Nm/A)	1.6
Motor Inertia $J_{m}$/($\mathrm{kg}m^{2}$)	7.17 × 10^−5^
Load Inertia $J_{l}$/($\mathrm{kg}m^{2}$)	7.31
Transmission Stiffness $K_{s}$/(Nm/rad)	50,000
Backlash Width/(arcm)	10
Encoder Resolution/($m^{-1}$)	223
Reduction Ratio of Harmonic Reducer	81
Cascade PI parameters $k_{p}$, $k_{vp}$, $k_{vi}$	32, 20, 80
Double Lopp Time Constant T/(s)	0.1
LESO Bandwidth/(rad/s)	3000
Solver	ode3 (Bogacki-Shampine)
Solver Step Size/(s)	1/16,000
Notch Filter Center Frequency (Natural Frequency)/(rad/s)	74.7699
Notch Filter Depth And Width parameters	2.5 & 10

## Data Availability

The data presented in this study are available on request from the corresponding author. The data are not publicly available due to commercial confidentiality restrictions of Shenzhen Inovance Technology Co., Ltd.
